# Cranial Nerve Palsies: Sarcoidosis to Systemic Lupus Erythematosus

**DOI:** 10.1155/2013/175261

**Published:** 2013-01-22

**Authors:** Fawad Aslam, Firas Bannout, Elizabeth B. Russell

**Affiliations:** ^1^Division of Rheumatology, Department of Internal Medicine, University of Arkansas for Medical Sciences, Little Rock, AR 72205, USA; ^2^Division of Rheumatology, Department of Internal Medicine, John L. McClellan Memorial Veterans Hospital, Little Rock, AR 72205, USA; ^3^Department of Neurology, University of Arkansas for Medical Sciences, Little Rock, AR 72205, USA

## Abstract

Cranial palsies are a very rare feature of SLE. Similarly, peripheral sensory-motor axonal neuropathy is very uncommon in SLE. The combination of the two as the presenting symptoms of SLE is a diagnostic challenge particularly in an elderly male patient with a known diagnosis of sarcoidosis. This case serves to highlight the diagnostic considerations in such a patient. The lack of response to standard therapy and the presence of subtle clues like anemia, proteinuria, and mild serositis should prompt the physician to look for alternate diagnoses. The potential association of SLE and sarcoidosis is also discussed. SLE can be present in elderly male patients with cranial and peripheral neuropathy.

## 1. Introduction

Central nervous system (CNS) involvement in systemic lupus erythematosus (SLE) is approximated at 50% and ranges from 14 to 75% [1]. Cranial palsies are an unusual central nervous system manifestation of SLE and account for 0.5–1.0% of neuropsychiatric manifestations [2]. Most frequent cranial neuropathies involve the eighth nerve followed by the oculomotor set (third, fourth, and sixth), and then the fifth and seventh nerves [3]. In sarcoidosis, 10% patients develop CNS involvement with 50–75% of cases having cranial involvement, most commonly the facial nerve [4]. Of anecdotal interest, SLE is the most frequent connective tissue disease associated with sarcoidosis [5] and the two share some underlying pathophysiological mechanisms [6]. We report a case of multiple cranial nerve palsies and subacute progressive, symmetric peripheral neuropathy simulating Guillain-Barre syndrome (GBS) in a patient with sarcoidosis who was eventually diagnosed with SLE.

## 2. Case Report

A 61-year-old African-American male presented to the emergency room with a few days history of double vision, left eyelid droop, and numbness and tingling in both hands and feet. He also endorsed one month history of profound fatigue and a progressive weakness with overhead movements and climbing stairs. Now to the point he was unable to ambulate and a recent fall triggered his visit.

 He had a history of biopsy proven sarcoidosis in 1970. He was treated with steroids for one year. He had gout, dyspepsia, and mild obstructive pulmonary disease. His medications included an albuterol inhaler, allopurinol, and omeprazole. He reported a weight loss of 30 pounds in the preceding six months. He had a chronic dry cough. 

**Figure 1:**
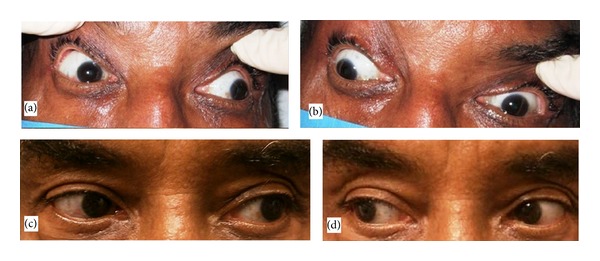
Pre-treatment leftward gaze (a), pre-treatment rightward gaze (b), post-treatment leftward gaze (c), post-treatment rightward gaze (d).

 Physical examination revealed a thin man with normal vital signs. Pertinent findings on exam were a significant ptosis of the left eye with only 1 mm aperture, an inability to fully adduct the left eye and inability to abduct the left eye (Figure 1), and an inability to achieve upward gaze in either eye. This was interpreted as partial right III and VI and almost complete left III and VI cranial neuropathies. There was subtle bilateral facial weakness and mild decrease of left facial sensation to pinprick test. He had proximal muscular atrophy and proximal weakness of both upper and lower extremities with 4/5 upper extremity strength and 3/5 lower extremities. Distal strength was preserved. He reported minimal tenderness on muscle palpation. Reflexes were decreased to absent bilaterally and sensation was somewhat decreased in the distal legs. His abdominal exam revealed slight tenderness with palpation of the entire abdomen. Joint examination was normal.

Admission labs revealed hemoglobin of 10.9 g/dL. Studies including calcium, electrolytes, creatinine, human immunodeficiency virus antibodies, vitamin B12, folate, creatinine protein kinase, aldolase, rapid plasma reagin, thyroid stimulating hormone, hepatitis screen, amylase, and lipase were normal except for mildly elevated AST and total bilirubin. His erythrocyte sedimentation rate (ESR) was elevated at 66 mm/hr (normal 0–40) while his C-reactive-protein (CRP) was normal. His urinalysis showed 3+ proteinuria that was normal 6 months ago. A computed tomography (CT) scan of the head was normal. Based on the initial impression of progressive symmetrical sensorimotor polyneuropathy and multiple cranial neuropathies, simulating GBS-like picture or a Miller-Fisher variant, the patient was treated with inpatient five days of IVIG. A working diagnosis of neurosarcoidosis, paraneoplastic syndrome, and vasculitic neuropathy was entertained.

A CT of the chest confirmed multiple calcified nodules with some increase in size and a small new right lower lobe infiltrate. Magnetic resonance imaging (MRI) and magnetic resonance angiogram (MRA) of the brain (including MRI orbit with and without gadolinium injection) were normal except for a small incidental aneurysm of distal right MCA branch. An electromyographic (EMG) and nerve conductive study (NCS) revealed a diffuse, predominantly axonal sensory-motor length-dependent polyneuropathy and myopathy with some suggestion of a mononeuritis multiplex undergoing generalized transformation. MRI of the lumbosacral spine showed multilevel degenerative changes. A spinal tap including cytology was negative except for elevated protein at 105 mg/dL (albumin-cytologic dissociation). 

Workup was done for a paraneoplastic etiology. CT abdomen was normal except a small amount of free fluid in the pelvis. Prostate-specific antigen colonoscopy was normal. A fine needle aspiration biopsy of the lung infiltrate revealed an inflammatory infiltrate with increased plasma cells but no granulomas and all cultures were negative. A muscle biopsy revealed moderately severe inflammatory myopathy with muscle fiber atrophy, regenerating fibers, and few ragged red fibers with no evidence of vasculitis. Endomysial fibrosis was also seen. A Sural nerve biopsy was negative for any acute abnormality but revealed evidence of possible old vasculitis. 

Lack of response to five doses of IVIG and a positive antinuclear antibody (ANA) (>1 : 640) prompted a rheumatology consultation on day 7 of his admission. Anti-SSA was strongly positive (>8.0) but other antibodies were negative. Complements were normal. Anticardiolipin, beta-2 glycoprotein-1-antibodies, lupus anticoagulant, and antineutrophil cytoplasmic antibodies were negative. Anti-Hu, anti-Ri, anti-YO, MUSK, antivoltage-gated calcium channel, acetylcholine receptor antibodies, Gq1b, angiotensin converting enzyme, lyme antibodies were negative. A 24 hour urine showed 1.1 gram protein. Serum protein electrophoresis showed slightly increased polyclonal immunoglobulin G (IgG). An echocardiogram revealed small pericardial effusion. On day 12 of admission, patient's Hb dropped to 7 g/dL without any triggering etiology. Serum haptoglobin was decreased and direct/indirect Coombs were positive. He was treated with pulse steroids for 5 days and then oral prednisone thereafter with improvement in his anemia but unchanged neurological status. 

 Based on the presence of autoantibodies, Coombs positive hemolytic anemia, proteinuria, myositis, serositis as manifested by mild abdominal pain, pelvic free fluid, and small pericardial effusion, symmetrical progressive sensorimotor polyneuropathy simulating GBS and multiple cranial neuropathies, he was diagnosed with SLE. He was begun on IV cyclophosphamide (CYC) approximately 3 weeks after presentation and had very significant improvement in both motor strength and extraocular movement within 4 weeks (Figure 1). He has presently received six infusions of cyclophosphamide and is off the prednisone and doing well on methotrexate. He is undergoing rehabilitation and has had complete resolution of his diplopia and anemia. His serositis and proteinuria also resolved. 

## 3. Discussion

This patient's presentation with cranial neuropathies and axonal peripheral neuropathies were initially felt to be a primary neurologic syndrome and sarcoidosis was the initial concern given his past history. However, the absence of active sarcoidosis on lung, muscle, and nerve biopsy, lack of any leptomeningeal enhancement or white matter lesions on brain MRI suggestive of sarcoidosis, and lack of response to steroids argued against sarcoidosis. Moreover presence of autoantibodies, autoimmune hemolytic anemia, proteinuria, serositis, and evidence of old vasculitis were suggestive of SLE. No occult cancer was identified although this was a major concern. The response to CYC was further convincing of the autoimmune diagnosis. 

Ocular manifestations in SLE are fairly common and estimated to occur in up to 60% of patients [7]. However, ocular motor abnormalities are infrequent. Most reported ocular motor abnormalities in SLE are isolated findings but, as in our case, multiple oculomotor cranial neuropathies have been described [8]. 

Peripheral neuropathy in SLE is predominantly of the sensory variety however, in one study of 1533 SLE patients, 13.5% had some peripheral neuropathic involvement of which only 18.8% were of the sensory-motor type [9]. Usually peripheral neuropathy appears in the established SLE cases, and rarely as the initial presentation. Polyneuropathy in itself is an uncommon presentation of SLE and is responsible for only 2.0-3.0% of all neuropsychiatric SLE cases [2].

 The pathogenic mechanism of cranial neuropathies in the setting of SLE is often difficult to verify. Vasculitis is often suspected because the patient has evidence of vasculitis elsewhere. In our patient, there was some evidence of healed vasculitis. Pathologic antibodies have been demonstrated to cause neurotoxicty or demyelination [10]. Another postulated mechanism is damage by local production of inflammatory cytokines [11]. Lastly, antiphospholipid antibodies have been associated with central nervous system disease [12] and the mechanism is assumed to be due to neural ischemia from microangiopathic thrombi. As a general rule, focal symptoms may be attributed to a thrombotic state in the right clinical settings while more generalized symptoms may be due to antibody or inflammatory etiologies [2]. 

Treatment of cranial nerve palsies in the setting of lupus involves immunosuppression. In cranial SLE, CYC, and intravenous methylprednisone are recommended by published guidelines [3]. An open-label study with rituximab has shown good results in 10 SLE patients with refractory neuropsychiatric lupus [13]. There are reports of the use of IVIG, azathioprine, and plasmapaharesis. 

Coexistence of sarcoidosis and SLE has been described in the literature. In one study of biopsy proven sarcoidosis patients, about 29% were positive for ANA [14]. Both diseases share certain features like B and T cell abnormalities and hypergammaglobulinemia, [5, 6]. The exact relationship between the two, if any, remains to be elucidated. Our case was also unique because most cases of co-existent SLE and sarcoidosis have been reported in females [5, 15].

In conclusion, we present a patient with cranial and peripheral neuropathies with a history of sarcoidosis who was eventually diagnosed with SLE and responded well to CYC treatment. SLE should be retained as a differential for cranial neuropathy in patients with known sarcoidosis. 

## References

[1] Bruns A, Meyer O (2006). Neuropsychiatric manifestations of systemic lupus erythematosus. *Joint Bone Spine*.

[2] Bertsias GK, Boumpas DT (2010). Pathogenesis, diagnosis and management of neuropsychiatric SLE manifestations. *Nature Reviews Rheumatology*.

[3] Bertsias GK, Ioannidis JPA, Aringer M (2010). EULAR recommendations for the management of systemic lupus erythematosus with neuropsychiatric manifestations: report of a task force of the EULAR standing committee for clinical affairs. *Annals of the Rheumatic Diseases*.

[4] Joseph FG, Scolding NJ (2007). Sarcoidosis of the nervous system. *Practical Neurology*.

[5] Enzenauer RJ, West SG (1992). Sarcoidosis in autoimmune disease. *Seminars in Arthritis and Rheumatism*.

[6] Veien NK, Hardt F, Bendixen G (1976). Immunological studies in sarcoidosis: a comparison of disease activity and various immunological parameters. *Annals of the New York Academy of Sciences*.

[7] Davies JB, Rao PK (2008). Ocular manifestations of systemic lupus erythematosus. *Current Opinion in Ophthalmology*.

[8] Keane JR (1995). Eye movement abnormalities in systemic lupus erythematosus. *Archives of Neurology*.

[9] Florica B, Aghdassi E, Su J, Gladman DD, Urowitz MB, Fortin PR (2011). Peripheral neuropathy in patients with systemic lupus erythematosus. *Seminars in Arthritis and Rheumatism*.

[10] Omdal R, Brokstad K, Waterloo K, Koldingsnes W, Jonsson R, Mellgren SI (2005). Neuropsychiatric disturbances in SLE are associated with antibodies against NMDA receptors. *European Journal of Neurology*.

[11] Dellalibera-Joviliano R, Dos Reis ML, Queiroz Cunha FD, Donadi EA (2003). Kinins and cytokines in plasma and cerebrospinal fluid of patients with neuropsychiatric lupus. *Journal of Rheumatology*.

[12] Meroni PL, Tincani A, Sepp N (2003). Endothelium and the brain in CNS lupus. *Lupus*.

[13] Tokunaga M, Saito K, Kawabata D (2007). Efficacy of rituximab (anti-CD20) for refractory systemic lupus erythematosus involving the central nervous system. *Annals of the Rheumatic Diseases*.

[14] Weinberg I, Vasiliev L, Gotsman I (2000). Anti-dsDNA antibodies in sarcoidosis. *Seminars in Arthritis and Rheumatism*.

[15] Begum S, Li C, Wedderburn LR, Blackwell V, Isenberg DA (2002). Concurrence of sarcoidosis and systemic lupus erythematosus in three patients. *Clinical and Experimental Rheumatology*.

